# RGB-to-Infrared Translation Using Ensemble Learning Applied to Driving Scenarios

**DOI:** 10.3390/jimaging11070206

**Published:** 2025-06-20

**Authors:** Leonardo Ravaglia, Roberto Longo, Kaili Wang, David Van Hamme, Julie Moeyersoms, Ben Stoffelen, Tom De Schepper

**Affiliations:** 1 Interuniversity Microelectronics Centre, Kapeldreef 75, 3001 Leuven, Belgiumtom.deschepper@imec.be (T.D.S.); 2 Image Processing and Interpretation, Ghent University, 9000 Ghent, Belgium

**Keywords:** machine learning, image processing, data augmentation, autonomous driving

## Abstract

Multimodal sensing is essential in order to reach the robustness required of autonomous vehicle perception systems. Infrared (IR) imaging is of particular interest due to its low cost and complementarity with traditional RGB sensors. However, the lack of IR data in many datasets and simulation tools limits the development and validation of sensor fusion algorithms that exploit this complementarity. To address this, we propose an augmentation method that synthesizes realistic IR data from RGB images using gradient-boosting decision trees. We demonstrate that this method is an effective alternative to traditional deep learning methods for image translation such as CNNs and GANs, particularly in data-scarce situations. The proposed approach generates high-quality synthetic IR, i.e., Near-Infrared (NIR) and thermal images from RGB images, enhancing datasets such as MS2, EPFL, and Freiburg. Our synthetic images exhibit good visual quality when evaluated using metrics such as $R^{2}$, PSNR, SSIM, and LPIPS, achieving an $R^{2}$ of 0.98 on the MS2 dataset and a PSNR of 21.3 dB on the Freiburg dataset. We also discuss the application of this method to synthetic RGB images generated by the CARLA simulator for autonomous driving. Our approach provides richer datasets with a particular focus on IR modalities for sensor fusion along with a framework for generating a wider variety of driving scenarios within urban driving datasets, which can help to enhance the robustness of sensor fusion algorithms.

## 1. Introduction

Autonomous vehicles are expected to revolutionize transportation systems, offering significant benefits such as improved safety, improved road efficiency, greater accessibility, and reduced environmental impact. These vehicles are expected to play a key role in the future of urban mobility, addressing challenges in traffic management and providing a safer and more sustainable alternative to traditional transportation systems [1,2]. However, achieving fully autonomous driving (level-5 capabilities) remains a great challenge which requires robust solutions to navigate complex scenarios [3]. One of the bottlenecks for achieving this level of autonomy is the robustness of the perception system. The vehicle must be able to perceive hazards and obstacles in all environments and under all conditions. To satisfy this requirement, autonomous vehicle prototypes employ multimodal sensing systems in which different sensors provide redundancy and complementarity [4].

One sensor type that is of high interest for this scope consists of Infrared (IR) imagers, especially in the Near-Infrared (NIR) and thermal wavelengths. By covering an additional part of the light spectrum, these sensors can enhance the detectability of hazards on or near the road while building on the significant progress that has been made in image-based object detection over the past decade. However, few large datasets currently contain IR data, which obstructs the development and validation of machine learning-based sensor fusion algorithms designed to process such input. One practical way to address this data scarcity is by using image translation to augment existing datasets with synthetic IR data. Although algorithms should not exclusively rely on synthetic data for training, this augmentation strategy can greatly increase the volume of available data, allowing more robust validation of a model’s stability and generalization capacity.

In this work, we propose a novel approach to address these challenges and make the following primary contributions:

(I) We demonstrate the effectiveness of ensemble learning for RGB-to-NIR image translation using a limited amount of training data. This is done by accurately selecting input features in addition to RGB channels, such as horizontal/vertical gradients and segmentation masks. The main objective is to achieve good visual quality and performances comparable with translations implemented with more complex methods and reported in the literature.
(II) We extend the method to RGB-to-thermal translation, tackling the one-to-many mapping (as one RGB can generate several thermal images, depending on the temperature conditions) by introducing a recursive training method generating multiple checkpoints. Additionally, we develop a regularized loss function for gradient boosting that enhances regression by considering the physical constraints of key elements in the scene with the highest thermal information, specifically, pedestrians and cars.
(III) We apply both RGB-to-NIR and RGB-to-thermal translation to images generated with the CARLA simulator for autonomous driving, providing an essential tool for evaluating new modalities and testing fusion pipelines. Ultimately, we apply the regularized translation model to the CARLA-generated images.

The rest of this article is organized as follows. In Section 2, we present the state of the art supporting this work. In Section 3, we present the method; in particular, Section 3.1 presents the training datasets, consisting of subsets from various publicly available datasets. As an alternative to more complex deep learning methods, we translate RGB images to NIR and thermal images using gradient boosting (see Section 3.2). Section 4.1 illustrates the model’s ability to maintain essential features of input images while successfully adapting them to desired target styles or formats. In addition, comparisons with GAN-based models and typical metrics in regression/computer vision reported in the literature are also discussed. This section also presents additional results considering the regularized loss.

Finally, in Section 4.2 we apply the proposed methodology for infrared translation to synthetic RGB images generated with the CARLA autonomous driving simulator.

## 2. Background

Multi-sensor fusion, particularly the combination of RGB with NIR or thermal imaging, has become imperative for safety-critical applications in autonomous driving, as each of these modalities provides complementary information. In urban environments, subtle cues from thermal or NIR images significantly enhance pedestrian detection, collision avoidance, and road boundary estimation [5], thereby alleviating the shortcomings of observations based only on RGB [6]. For instance, NIR images capture distinct textural details, whereas thermal images highlight heat signatures, revealing objects or living beings otherwise hidden from standard RGB sensors. Such sensor fusion pipelines can robustly handle varying lighting conditions, including nighttime occlusions and adverse weather conditions, ultimately improving the understanding of the scene [7].

Deep learning has emerged as the gold standard for tackling sensor fusion approaches, primarily through the use of large Convolutional Neural Networks (CNNs) [8,9]. Due to the lack of real device data, sensor fusion can be supported by image translation techniques. These techniques were originally implemented for style and domain transfer with the aim of learning domain mappings while disentangling content and style features. The main paradigm for translation tasks is that of Generative Adversarial Networks (GANs), which have demonstrated success in various generation tasks and image translation to various styles [10,11,12,13,14]. GAN approaches rely on architectures such as Pix2Pix/Pix2PixHD [10,15], CycleGAN [11], DualGAN [12], DiscoGAN [13], and UNIT/MUNIT [14,16,17]. However, these methods often come with significant computational costs and are susceptible to challenges such as mode collapse and overfitting.

In our context, we focus on approaches that tackle modality translation, such as RGB-to-NIR/thermal, which are critical for enhancing cross-modal understanding and data augmentation. For example, Jin et al. [18] leveraged vision foundation models in their Pix2Next framework to achieve high-quality RGB-to-NIR translation, demonstrating the potential of pretrained models for this task. Yang et al. [19] addressed nighttime challenges by integrating visible images for thermal infrared translation, improving robustness in low-light conditions. Similarly, Jeon et al. [20] proposed RainSD, a module that enhances image synthesis through feature-level style diversification, which can also benefit cross-modal tasks. Zhai et al. [21] focused on spectral consistency with their ColorMamba model for NIR-to-RGB translation, ensuring high-quality output. These methods collectively support data augmentation, which is essential for feeding sensor fusion pipelines [22]. Additionally, thanks to these approaches, specific corner cases in urban driving can be addressed; for example, converting RGB images to thermal can address nighttime visibility problems [23] and offer improved recognition in harsh weather [7]. Ultimately, style transfer facilitates the training of more robust perception models thanks to enriched datasets with rain-distorted images [20,24]. Consequently, translation-based methods provide a scalable solution for enhancing existing multimodal datasets, making them more robust and diverse.

While deep learning remains the gold standard, its computational requirements and the inherent difficulties of collecting large training sets can represent an important drawback. Hence, lower complexity alternatives have been explored, such as simpler linear encoder–decoders with principal component analysis [25] and other decision tree-based methods [26]. These may underperform in highly nonlinear tasks, whereas use of the ensemble strategies presented later in this paper offers better performance and can mitigate overfitting in data-scarce scenarios. This is particularly crucial for real-world autonomous systems that must adapt to diverse and dynamically changing environments.

Finally, tree ensembles such as gradient boosting preserve instance–level interpretability because each decision node encodes an explicit human-readable binary rule; this contrasts with the distributed representations learned by deep CNN or GAN generators.

## 3. Method

### 3.1. Data Sources and Features Extraction

**Figure 1:**
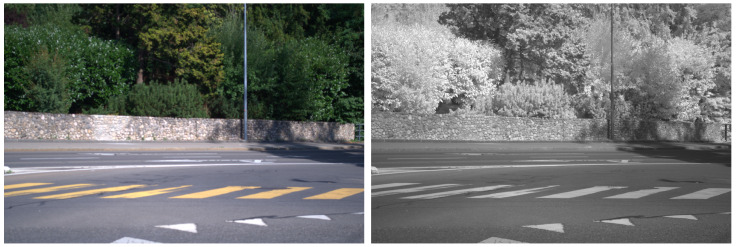
Example from the EPFL dataset: RGB (**left**) and NIR (**right**) pair [27].

In this work, three different datasets are used for image translation. Two of them (EPFL and MS2) are used to train and test different styles of NIR images, while the third (Freiburg) is used for thermal images. The EPFL dataset consists of 477 images in nine categories captured in RGB and NIR [27]. The images were captured using separate exposures from modified SLR cameras, using filters for both visible and NIR wavelengths. The scene categories include country, field, forest, indoor, mountain, old building, street, urban, and water. An example of RGB and NIR pairs that make up the training dataset is shown in Figure 1; the left side of the figure contains an RGB image extracted from the EPFL dataset, showing a road with signs and vegetation, while the right side contains the same image captured by the NIR sensor, where the vegetation shows high-intensity pixels typical of NIR images.

**Figure 2:**
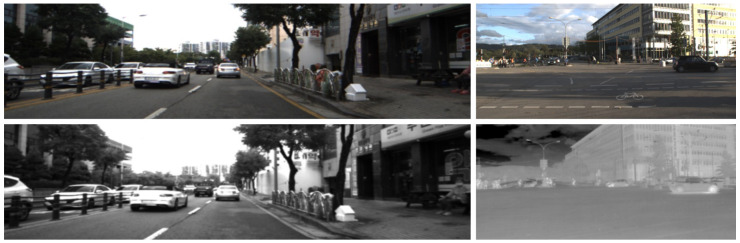
Examples of RGB/NIR images included in MS2 dataset [28] (**left side**) and RGB/thermal images from the Freiburg dataset [29] (**right side**).

On the other hand, the outdoor Multispectral Stereo (MS2) dataset includes stereo RGB, stereo NIR, stereo thermal, stereo LiDAR data, and GPS/IMU information [28]. This dataset provides 184,000 data pairs taken from city, residential, road, campus and suburban areas in the morning, daytime, and nighttime under clear skies, cloudy, and rainy conditions. The Freiburg dataset [29] consists of real RGB/thermal pairs captured in a driving context. The images displayed in Figure 2 include examples from the MS2 and Freiburg datasets, showing RGB, NIR, and thermal imagery. The left side of Figure 2 shows RGB and NIR data from the MS2 dataset, with the latter exhibiting significant differences such as darker vegetation compared to the EPFL data type. The right side shows examples from the Freiburg dataset, with the IR image (bottom) highlighting the thermal signatures of various objects, particularly car wheels and pedestrians crossing on the opposite side of the intersection.

Using these datasets, we created a feature matrix as input for the training procedure. The feature matrix was constructed such that each row corresponded to a pixel in the RGB domain. The resulting matrix consists of six columns, with each column representing a different feature; specifically, the features included in the matrix were the R, G, and B channels. In addition, we added horizontal and vertical gradients as features, providing insight into changes in intensity and direction within the image. Finally, to provide information about the different material properties of the objects in the scene, we included the class associated with each pixel through semantic segmentation, which was obtained using the Detectron2 model [30]. It is worth noting that considering features such as segmentation masks can facilitate domain transfer from real-world scenarios to a simulated environment. We acknowledge that the use of pretrained segmentation models increases the complexity of the proposed approach. We make the assumption that this resource is already available, as in many applications dedicated to autonomous driving. Moreover, the CARLA simulator (Section 4.2) provides perfect segmentation masks, helping the translation model to better understand the semantics of the input image. The entire process is ultimately supervised by the label vector containing the pixel values from the real NIR or thermal image.

### 3.2. Modeling and Training Procedure

**Figure 3:**
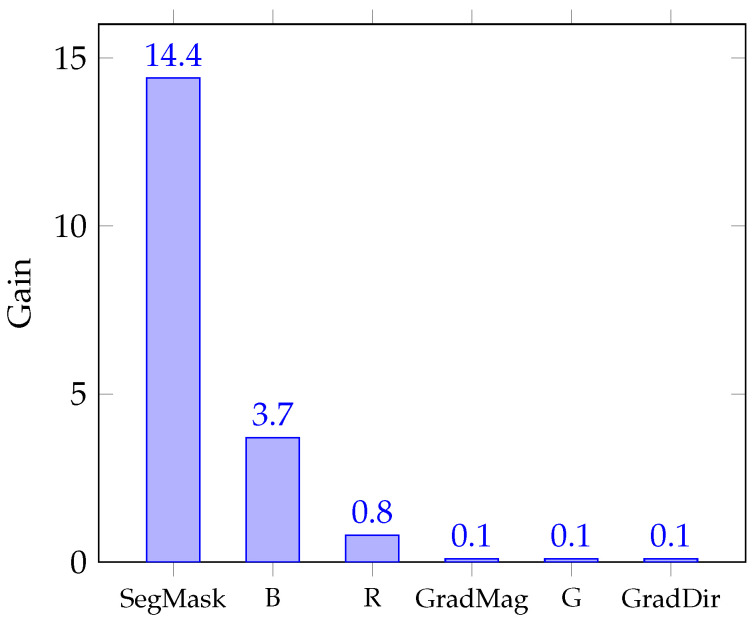
Gain-based feature importance obtained from the first XGBoost training batch.

We selected a gradient boosting model for this study. Gradient boosting belongs to the ensemble learning group of machine learning models, as already mentioned in the Introduction section. This choice was established after k-fold cross-validation (k=7) trials among different popular regression models such as multivariate linear regression, support vector machine, decision trees, and random forest. Gradient boosting combines weak learners with limited depth, such as decision trees, to create a more accurate predictor. It works by sequentially building simpler prediction models, with each model aiming to predict the errors left over from the previous model. The gain-based feature importance analysis shown in Figure 3 suggests that the segmentation mask dominates all other features, indicating that the infrared signature is largely object-dependent, whereas raw color and gradient information provides only marginal additional insight. The hyperparameters were set after a tuning process via cross-validation, leading to selection of number of trees = 300 and max tree depth = 10. The process of RGB-to-NIR translation is treated as a supervised regression problem at the pixel level.

For each dataset, a gradient boosting model was trained with the aim of reproducing the same style observed in the training data. For training image translation in EPFL style, we carefully selected 35 images from the street, urban, and country folders. These images showcased the best RGB/NIR time synchronization. On the other hand, the MS2 training subset contained 500 consecutive frames with a size of 700 px by 320 px. Both selections covered a wide range of weather and lighting conditions. The library used for the model implementation was XGBoost [31], with Python 3.9 as the main coding language and Pytorch v1.9 for computation of the metrics. The pseudo-code describing the feature matrix construction and the supervised gradient boosting regression training is reported in Algorithm 1.
**Algorithm 1:** Feature Matrix Construction and Training
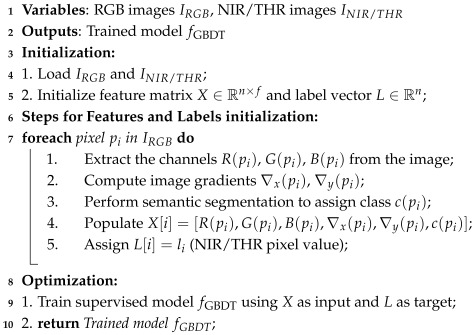


**Figure 4:**
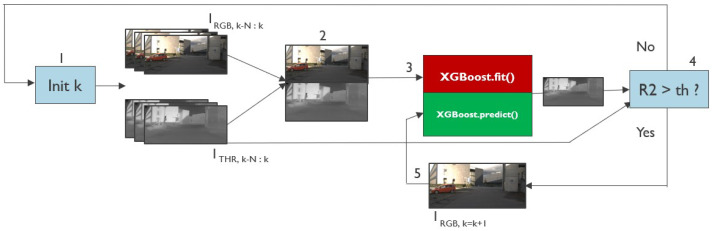
Flow chart of the iterative training algorithm for RGB-to-thermal translation.

Translating thermal data requires a more complex training process. As mentioned in the Introduction section, this complexity arises because the same RGB image can produce different thermal images depending on the temperature conditions. To improve the accuracy of temperature estimation in the considered driving scene, the pixel-to-pixel supervised regression for RGB-to-thermal translation is improved by simulating an iterative training situation, as illustrated in Figure 4. In detail, the training procedure involves the retrieval of N=10 consecutive frames of real RGB and thermal images during the vehicle motion (step 1 and step 2 in in Figure 4). This batch is used to train the gradient boosting model, as detailed in Algorithm 1, with the aim of predicting thermal images based on the acquired RGBs (step 3 in Figure 4). During the training process, the $R^{2}$ score is calculated over the validation portion, which constitutes 25% of the total batch of pixels (step 4 in Figure 4). If the $R^{2}$ coefficient surpasses a predetermined threshold (set in this case to 0.65), a new checkpoint is stored and the algorithm switches to test mode (step 5 in Figure 4). In test mode, predictions of synthetic thermal images based on the current RGB frame are generated. The process continues until the $R^{2}$ coefficient calculated between the current predicted thermal image and the last real thermal image belonging to the training dataset falls below the threshold. If this occurs, a new training cycle begins. This entails acquiring a new batch of data, again consisting of ten consecutive RGB and thermal images (step 1).

Furthermore, we extend the method to focus on the thermal signatures captured by the classes pedestrian (referring here to human bodies in general) and car by proposing a regularized loss that emphasizes pixels belonging to either of the two classes (the masked penalty term in Equation (1)). This customized loss also imposes constraints on predictions to keep them within a predefined range of values (the physical bounds penalty term in Equation (1)).

(1) $$L\;\left(\hat{y},y,m\right)=\underset{\mathrm{Residual}\;\mathrm{Loss}}{\underset{︸}{\sum_{i=1}^{N}{\left({\hat{y}}_{i}-y_{i}\right)}^{2}}}\;+\;\lambda \;\underset{\mathrm{Masked}\;\mathrm{Penalty}}{\underset{︸}{\sum_{i=1}^{N}P_{\mathrm{masked}}\;\left(i;m\right)}}\;+\;\gamma \;\underset{\mathrm{Physical}\;\mathrm{Bounds}\;\mathrm{Penalty}}{\underset{︸}{\sum_{i=1}^{N}P_{\mathrm{physical}}\;\left(i;m\right)}}$$

In detail, the masked penalty term in Equation (1) imposes a penalty on the normalized residuals for specific mask values:

(2) $$P_{\mathrm{masked}}\;\left(i;m\right)=\left\{\begin{array}{cc} {\left(\frac{{\hat{y}}_{i}-y_{i}}{\sigma_{{\left(m\right)}_{i}}+\epsilon }\right)}^{2\alpha }, & \mathrm{if}\;{\left(m\right)}_{i}=m\\ 0, & \mathrm{otherwise} \end{array}\right.$$

where:

- $\sigma_{{\left(m\right)}_{i}}=\sqrt{\mathrm{Var}\;\left(\{y_{j}:{\left(m\right)}_{j}=m\}\right)+\epsilon }$ is the standard deviation of the true labels for mask *m* (with ϵ for numerical stability).
- α is an additional hyperparameter (here, α=1).
- λ is the regularization coefficient (here, λ=1).

Conversely, the physical bounds penalty term ensures that predictions remain within the predefined range $\left[a_{m},b_{m}\right]$ for each mask *m*:

(3) $$P_{\mathrm{physical}}\;\left(i;m\right)=\left\{\begin{array}{cc} {\left(\max(a_{m}-{\hat{y}}_{i},0)\right)}^{2}+{\left(\max({\hat{y}}_{i}-b_{m},0)\right)}^{2}, & \mathrm{if}\;{\left(m\right)}_{i}=m\\ 0, & \mathrm{otherwise} \end{array}\right.$$

where:

- $a_{m}$ and $b_{m}$ are the lower and upper bounds for mask *m* for each class (e.g., person or vehicle) measured in the training set.
- γ is the regularization coefficient (here, γ=0.5).

## 4. Results and Discussion

### 4.1. Infrared Translated Images and Performance Comparisons

**Figure 5:**
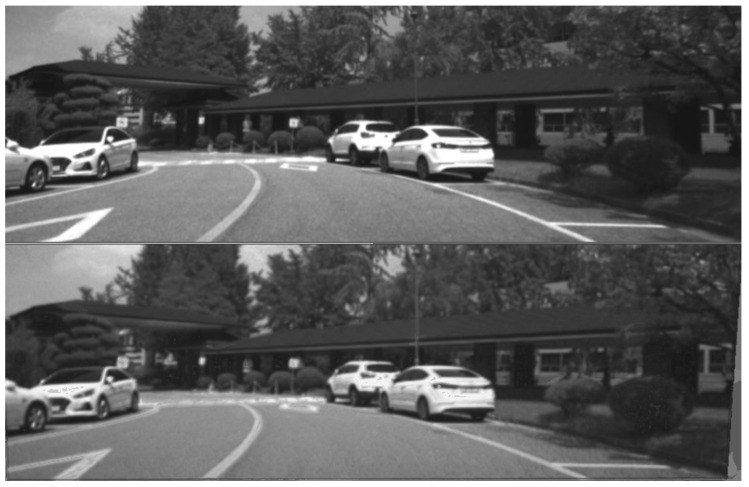
Ground truth (**top**) and synthetic NIR (**bottom** using the proposed method) images from MS2 dataset [28].

**Figure 6:**
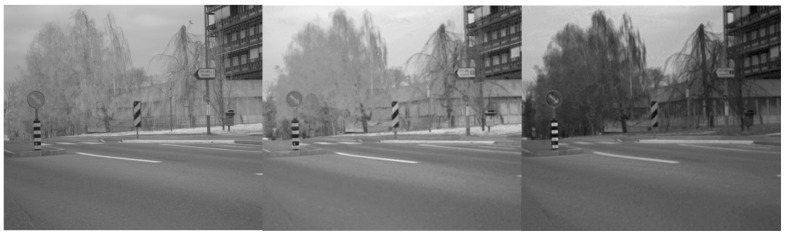
Ground truth [27] (**left**) and synthetic NIR images generated using the proposed method reproducing the EPFL (**center**) and MS2 (**right**) styles.

**Figure 7:**
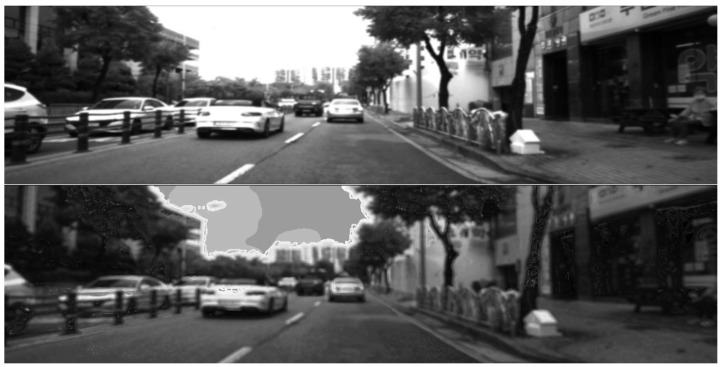
Ground truth (**top**) and synthetic NIR (**bottom** using the proposed method). The translated image exhibits artifacts because of the saturated sky pixel levels (MS2 dataset [28]).

In this section, we present the results of image translations across the different datasets. The images were processed as described in Section 3, transforming the original RGB data into synthetic NIR and thermal data. In Figure 5 and Figure 6, we report examples of NIR translation outcomes together with the original MS2 and EPFL images. Figure 5, presents the translation of the image using a model trained in the MS2 dataset, where smaller artifacts are present on vehicles and road signs but with a rather low impact on the global visual quality of the image. Moreover, Figure 6 shows the conversion of an EPFL RGB image to a synthetic NIR image applying the MS2 style. This figure showcases both types of translation; it is worth noticing the overall high visual quality in both of the outcomes. On the other hand, it is important to point out that translations can be negatively affected by the presence of saturated pixel levels in the RGB images (mainly occurring in the sky). An example is shown in Figure 7, where saturated pixels in the sky region are translated into artifacts in the corresponding synthetic NIR image (bottom figure).

**Figure 8:**
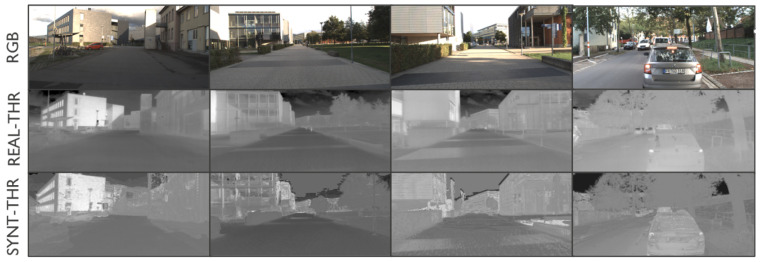
RGB, ground truth, and synthetically generated thermal images (Freiburg dataset [29]).

As already mentioned, the translation of thermal images is more complex, as the conversion should consider the temperature conditions related to every object in the acquired RGB images. For instance, in traffic scenarios, thermal imaging typically reveals higher temperatures in regions surrounding the wheels and engines of vehicles. As reported in Section 3.2, we apply a recursive training approach to mitigate these limitations. Examples results are shown in Figure 8. Even in this case, the quality of the translation is globally lower in saturated zones such as the sky. Nevertheless, zones with different temperature conditions can still be distinguished. Furthermore, Figure 8 illustrates the model’s ability to generalize across various urban streets. The first three columns show the performance of the model on the same street without requiring retraining, highlighting its robustness in similar environments. In contrast, the fourth column presents a situation in which the vehicle encounters a new street, necessitating retraining of the model to adapt to the different conditions.

In Table 1, we analyze the performance of the entire translation process in 160 test images for each dataset, reporting mean and standard deviation values for the determination coefficient $R^{2}$ [32], Peak Signal-to-Noise Ratio (PSNR), Structural Similarity Index Measure (SSIM) [33], and Learned Perceptual Image Patch Similarity (LPIPS) [34].

The overall quality of the regression is assessed using the $R^{2}$ coefficient and PSNR, which are straightforward to interpret due to their relationship with the Mean Square Error (MSE). The SSIM is more bound to perception, as it models the human visual system by focusing on structural information, luminance, and contrast. This aligns more closely with how humans perceive image quality, whereas the MSE and PSNR rely solely on pixel-wise differences. In addition to these metrics, LPIPS is particularly valuable for evaluating perceptual similarity. Unlike traditional metrics, LPIPS leverages deep neural networks to model human visual perception, making it more effective in capturing subtle differences in image quality that align with human judgment. In this way, it provides a quantifiable measurement of the overall quality of the translated outputs.

Nonetheless, several investigations have shown that conventional error–based figures can diverge markedly from what human viewers actually perceive in the thermal–infrared (TIR) band. In the seminal paper *Subjective Quality Assessment of Thermal Infrared Images* [35], 36 observers rated 450 image sequences acquired with uncooled sensors; the authors reported that after fixed-pattern noise or low-contrast artifacts appear, the monotonic relation between Mean Opinion Scores (MOS) and PSNR/SSIM quickly degrades. A later work, *Study of Subjective and Objective Quality Assessment of Infrared Compressed Images* [36], analyzed the same scenes after H.265 compression and found that below 0.5 bpp, the Spearman correlation of PSNR with MOS drops under 0.80, whereas perceptually-grounded indices that mix structural and contrast cues remain above 0.85. These findings underpin our decision to report LPIPS alongside PSNR and SSIM.

Furthermore, as these are relatively common metrics, they enable us to perform fair and consistent evaluations of the obtained results while facilitating comparisons with other methodologies. In our case, all the datasets presented high values for the global metric $R^{2}$, with the MS2 dataset achieving a near-perfect score of 0.98 and the highest PSNR score of 21.3 dB being achieved on the Freiburg dataset, as reported in Table 1. For the Freiburg dataset, SSIM performance close to that obtained using deep learning models was achieved, as shown in the GAN (sRGB-TIR) column of Table 1. In more detail, it can be observed that the proposed XGBoost method performed even better than the GAN in terms of the LPIPS metric. For this first comparison we only used RGB-to-thermal translations, as to the best of our knowledge pretrained deep learning models for translating RGB-to-NIR modalities are not yet available. An additional comparison takes into account the Ref. Range column in Table 1, which reports typical values obtained by means of more complex architectures trained on larger-scale datasets. As mentioned, in our case the values of these metrics can be impaired by saturated pixel levels in the original RGB images (mainly in the sky and buildings). This issue can also explain the differences when compared with these reference values.

**Figure 9:**
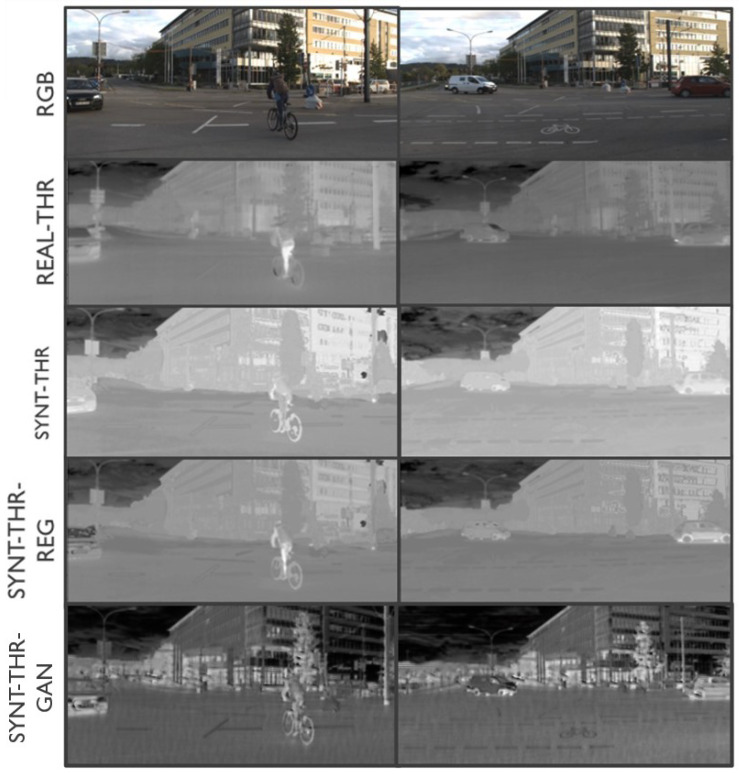
RGB, ground truth, and synthetic thermal images (without and with regularization) generated on test samples, showing both pedestrians and cars (Freiburg dataset [29]). The bottom row shows the translations obtained using the pretrained sRGB-TIR GAN model [37].

On the other hand, the regularized loss function in Equation (1) provides better contrast and more accurate temperature zones for pedestrians and cars in the scene, as depicted in Figure 9. For cars, the regularized loss particularly enhances the delineation of regions with higher temperature variations, such as areas adjacent to the wheels and engine, where it helps to reduce artifacts and preserve fine details. Table 2 reports improvements in regression metrics by comparing masked instances for pedestrians and cars, with an increase of up to 0.10 in the $R^{2}$ score and up to 0.70 dB for the PSNR on the pedestrians class. Overall, these improvements underscore the potential of integrating instance-specific and physical information into the loss components.

**Table 1:** Comparison of results between translations using the proposed XGBoost method on the EPFL, MS2, and Freiburg datasets (160 samples), including a GAN baseline evaluated on Freiburg and reference values. The mean and standard deviation are shown in brackets.

Metric	XGBoost			GAN (sRGB-TIR)	Ref. Range
	EPFL	MS2	Freiburg	Freiburg	
**$R^{2}$ (↑)**	0.94 (0.03)	**0.98 (0.003)**	0.86 (0.15)	0.85 (0.20)	>0.85 [38]
**PSNR (↑)**	18.7 (2.57)	17.1 (1.84)	21.3 (5.08)	**23.55 (3.14)**	>20 [39]
**SSIM (↑)**	0.54 (0.08)	0.62 (0.05)	0.75 (0.06)	**0.81 (0.05)**	>0.70 [40]
**LPIPS (↓)**	0.094 (0.04)	0.15 (0.08)	**0.038 (0.01)**	0.25 (0.03)	<0.1 [39]

**Table 2:** Performance gains of the regularized loss model over the standard loss model on thermal images. In particular, gains are reported for the Pedestrian and Car instances after training with the physically constrained penalties in Equation (1). The gain results highlight the contribution of regularization to the training process.

Metric	Pedestrian	Car
$R^{2}$	+0.10	+0.06
PSNR	+0.70 dB	+0.284 dB

### 4.2. Infrared Translation Using Synthetic RGB Images Generated in CARLA Simulator

In this section, we show the translation results obtained by using RGB images generated with CARLA (version 0.9.15) [41]. This platform is an open-source simulator for autonomous driving that is capable of handling various urban environments through a robust Python API, allowing for the simulation of different topographies, weather conditions, lighting, and actors (e.g., vehicles and pedestrians). This allows testing of a wide range of sensors in specific corner cases. The hardware platform used for both the simulation and the machine learning workloads was a virtual machine with Windows 11, an NVIDIA T4 GPU (NVIDIA, Santa Clara, CA, USA), and 32 GB of RAM. In this application, the primary goal of translating RGB CARLA images is to provide a quick alternative to developing the specific sensors within the simulator itself. A secondary goal is to use the generated images as a means of data augmentation to improve the training of data fusion algorithms like the ones proposed in [42,43].

**Figure 10:**
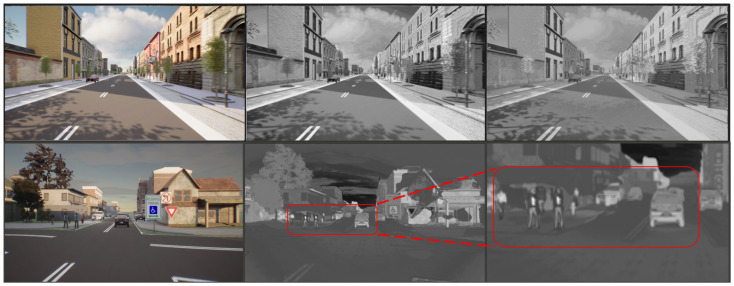
Comparison of simulated images in CARLA. **Top row**: RGB to NIR-MS2 style and NIR-EPFL style. **Bottom row**: RGB to thermal. The bottom right corner shows a zoomed-in version of the center-bottom image, highlighting the brighter thermal signature in the pedestrians and at the bottom of the vehicle.

Synthetic NIR images were generated using inferences from pretrained models with the MS2 and EPFL datasets. On the other hand, the RGB-to-thermal translations were produced by using one of the checkpoints obtained during recursive training on the Freiburg data with the regularized loss. As previously mentioned, we used the segmentation masks directly generated in CARLA because of their high quality. The results are depicted in Figure 10, where the differences between the two NIR styles (for example, in vegetation) can be observed. Similarly, the thermal version of the RBG image shows brighter signatures on pedestrians and at the bottom of vehicles.

## 5. Conclusions

This work has presented a method for translating RGB images to Infrared (IR) images by using an ensemble learning approach with a focus on NIR and thermal wavelengths. The proposed method includes segmentation masks as features for the regression problem, assuming their availability in most autonomous driving applications. Performance metrics such as the determination coefficient $R^{2}$, Peak Signal-to-Noise Ratio (PSNR), Structural Similarity Index Measure (SSIM), and Learned Perceptual Image Patch Similarity (LPIPS) are presented and compared with literature values obtained using gold-standard techniques (i.e., GANs); refer to Table 1 for the results. In particular, by using 35 non-consecutive images for training the EPFL camera model and 500 consecutive images for the MS2 camera model, we achieved image quality metrics comparable to state-of-the-art deep learning models, which are typically trained on many thousands of images (e.g., the GAN in [39] was trained on 21,000 to 170,000 images). However, our model showed lower metrics due to artifacts, which were mainly located in the sky and building areas of the images. Concerning thermal images, our custom regularized loss produced synthetic images of better quality compared with the standard ones for both the real and CARLA domains. Overall, the shortcomings of the proposed approach are inherently due to the low amount of training data; therefore, a dataset that contains an extensive representation of all the main road actors is fundamental for the training process.

In future work, we will investigate further integration and refinement of the model within autonomous driving simulation environments such as CARLA in order to broaden its practical application. In addition, we will adapt the models to simulate other parts of the infrared spectrum and explore other application domains, such as security and surveillance systems. In later work, we will explore alternative lightweight models that can support translation by refining the outputs and optimizing the perceived visual quality. 

## Data Availability

The raw data generated and supporting the conclusions of this article will be made available by the authors on request.

## Appendix A. Computational Complexity and Runtime

The translation pipeline has three stages: feature extraction, supervised training of the Gradient-Boosting Decision Tree (GBDT) ensemble model, and per-frame inference. Let the input image have resolution H×W, let f=6 be the feature vector length (Section 3.1), and let the ensemble contain T=300 trees of depth d=10.

Feature Extraction: Step 7 of Algorithm 1 scans every pixel one time in order to: (i) copy the three RGB channels, (ii) compute two Sobel gradients, and (iii) look up the semantic class. Hence, the time and memory costs are Θ(H×W) and Θ(H×Wf), respectively.

Training: XGBoost employs the approximate quantile sketch from [44]. Building one tree requires

$$O\;\left(\;f\;n\log n\;\right),\;n=HW$$

operations. With *T* and *f* fixed, the whole ensemble scales as

$$O\;\left(Tfn\log n\right),$$

i.e., quasi-linearly in the number of pixels.

Inference: Each pixel traverses at most *d* internal nodes in each of the *T* trees, for a cost of

$$O\;\left(TdHW\right).$$

Because *T* and *d* are constants, inference is strictly linear in the image size.

Wall-Clock Measurements: Table A1 provides the throughputs measured on a system with an Intel i7 CPU (Intel, Santa Clara, CA, USA) and NVIDIA T4 GPU. One full-HD frame (1920×1080) contains 7.8× more pixels than a 720×320 frame; the empirical ratios 4.3→0.5fps (CPU) and 4.3→0.5fps (GPU) agree closely with that prediction.

**Table A1:** Empirical run-time of the proposed GBDT ensemble.

Resolution	Throughput [fps]	
	CPU (i7-1185G7)	GPU (T4)
720×320	4.3	4.3
1920×1080	0.5	0.5

Memory Footprint: The serialized ensemble occupies only 5.6 MB. During streaming, the feature buffer for one 720×320 frame is 5.1 MB; when stored as a NumPy mem-map, RAM usage stays flat irrespective of video length.

Comparison with GAN Baseline: For parity, we performed a comparison against the lighter of the two directions of the *GAN* encoder–-decoder that performs the actual RGB→IR translation. Discriminators and gradient buffers were absent at test time, so only the generator branch was loaded.

(a) **Asymptotic cost:** A forward pass consists of L=13 convolutions, for which the arithmetic grows linearly with the number of pixels:
  $$O\;\left(PHW\right),\;P=10.5\;M\;\mathrm{learnable}\;\mathrm{weights}.$$
  Empirically, this is ≈15GFLOPs for one 720×320 frame, versus the $O\;\left(TdHW\right)$ integer comparisons used by the GBDT.
(b) **Memory footprint:** The FP32 weights occupy 40 MB, the peak activation tensor for a 720×320 input is 13 MB, and the cuDNN scratch space requires 250 MB. Hence, the entire translation branch fits comfortably within 303 MB of GPU RAM, compared with 5.6 MB for the serialized XGBoost ensemble and 5.1 MB for one feature buffer.

The GAN achieves an order-of-magnitude higher per-frame compute and memory load but can still run in real time on commercial GPUs, whereas the lightweight GBDT can be deployed efficiently on the CPU alone.

## References

[1] Pendleton S., Andersen H., Du X., Shen X., Meghjani M., Eng Y., Rus D., Ang M. (2017). Perception, planning, control, and coordination for autonomous vehicles. Machines.

[2] Adnan N. (2024). Exploring the future: A meta-analysis of autonomous vehicle adoption and its impact on urban life and the healthcare sector. Transp. Res. Interdiscip. Perspect..

[3] Sana F., Azad N.L., Raahemifar K. (2023). Autonomous vehicle decision-making and control in complex and unconventional scenarios—A review. Machines.

[4] Huang K., Shi B., Li X., Li X., Huang S., Li Y. (2022). Multi-modal sensor fusion for auto driving perception: A survey. arXiv.

[5] Marnissi M.A., Fradi H., Sahbani A., Amara N.E.B. (2021). Thermal image enhancement using generative adversarial network for pedestrian detection. Proceedings of the 2020 25th International Conference on Pattern Recognition (ICPR).

[6] Geronimo D., Lopez A.M., Sappa A.D., Graf T. (2009). Survey of pedestrian detection for advanced driver assistance systems. IEEE Trans. Pattern Anal. Mach. Intell..

[7] Aloufi N., Alnori A., Basuhail A. (2024). Enhancing Autonomous Vehicle Perception in Adverse Weather: A Multi Objectives Model for Integrated Weather Classification and Object Detection. Electronics.

[8] Fadadu S., Pandey S., Hegde D., Shi Y., Chou F.C., Djuric N., Vallespi-Gonzalez C. Multi-view fusion of sensor data for improved perception and prediction in autonomous driving. Proceedings of the IEEE/CVF Winter Conference on Applications of Computer Vision.

[9] Liu Z., Tang H., Amini A., Yang X., Mao H., Rus D.L., Han S. (2023). Bevfusion: Multi-task multi-sensor fusion with unified bird’s-eye view representation. Proceedings of the 2023 IEEE International Conference on Robotics and Automation (ICRA).

[10] Isola P., Zhu J., Zhou T., Efros A. Image-to-image translation with conditional adversarial networks. Proceedings of the IEEE Conference on Computer Vision and Pattern Recognition.

[11] Zhu J., Park T., Isola P., Efros A. Unpaired image-to-image translation using cycle-consistent adversarial networks. Proceedings of the IEEE International Conference on Computer Vision.

[12] Yi Z., Zhang H., Tan P., Gong M. DualGAN: Unsupervised dual learning for image-to-image translation. Proceedings of the IEEE International Conference on Computer Vision.

[13] Kim T., Cha M., Kim H., Lee J., Kim J. Learning to discover cross-domain relations with generative adversarial networks. Proceedings of the International Conference on Machine Learning, PMLR.

[14] Friedjungová M., Vašata D., Chobola T., Jiřina M. Unsupervised Latent Space Translation Network. Proceedings of the European Symposium on Artificial Neural Networks.

[15] Wang T., Liu M., Zhu J., Tao A., Kautz J., Catanzaro B. High-resolution image synthesis and semantic manipulation with conditional GANs. Proceedings of the IEEE Conference on Computer Vision and Pattern Recognition.

[16] Liu M., Breuel T., Kautz J. (2017). Unsupervised image-to-image translation networks. Adv. Neural Inf. Process. Syst..

[17] Huang X., Liu M.Y., Belongie S., Kautz J. Multimodal unsupervised image-to-image translation. Proceedings of the European Conference on Computer Vision (ECCV).

[18] Jin Y., Park I., Song H., Ju H., Nalcakan Y., Kim S. (2024). Pix2Next: Leveraging Vision Foundation Models for RGB to NIR Image Translation. arXiv.

[19] Yang S., Sun M., Lou X., Yang H., Liu D. (2024). Nighttime Thermal Infrared Image Translation Integrating Visible Images. Remote Sens..

[20] Jeon H., Seo J., Kim T., Son S., Lee J., Choi G., Lim Y. (2023). RainSD: Rain Style Diversification Module for Image Synthesis Enhancement using Feature-Level Style Distribution. arXiv.

[21] Zhai H., Jin G., Yang X., Kang G. (2024). ColorMamba: Towards High-quality NIR-to-RGB Spectral Translation with Mamba. arXiv.

[22] Wang Z., Colonnier F., Zheng J., Acharya J., Jiang W., Huang K. Tirdet: Mono-modality thermal infrared object detection based on prior thermal-to-visible translation. Proceedings of the 31st ACM International Conference on Multimedia.

[23] Liu S., Gao M., John V., Liu Z., Blasch E. (2020). Deep learning thermal image translation for night vision perception. ACM Trans. Intell. Syst. Technol. (TIST).

[24] Pizzati F., Charette R.d., Zaccaria M., Cerri P. Domain bridge for unpaired image-to-image translation and unsupervised domain adaptation. Proceedings of the IEEE/CVF Winter Conference on Applications of Computer Vision.

[25] Richardson E., Weiss Y. The surprising effectiveness of linear unsupervised image-to-image translation. Proceedings of the 2020 25th International Conference on Pattern Recognition.

[26] Lu K., Yang D. (2009). Image processing and image mining using decision trees. J. Inf. Sci. Eng..

[27] Brown M., Süsstrunk S. Multispectral SIFT for Scene Category Recognition. Proceedings of the Computer Vision and Pattern Recognition (CVPR11).

[28] Shin U., Park J., Kweon I.S. Deep Depth Estimation From Thermal Image. Proceedings of the IEEE/CVF Conference on Computer Vision and Pattern Recognition.

[29] Vertens J., Zürn J., Burgard W. (2020). HeatNet: Bridging the Day-Night Domain Gap in Semantic Segmentation with Thermal Images. arXiv.

[30] Wu Y., Kirillov A., Massa F., Lo W., Girshick R. (2019). Detectron2. https://github.com/facebookresearch/detectron2.

[31] Chen T., Guestrin C. XGBoost Documentation. https://xgboost.readthedocs.io.

[32] Chicco D., Warrens M.J., Jurman G. (2021). The coefficient of determination R-squared is more informative than SMAPE, MAE, MAPE, MSE and RMSE in regression analysis evaluation. PeerJ Comput. Sci..

[33] Wang Z., Bovik A., Sheikh H., Simoncelli E. (2004). Image quality assessment: From error visibility to structural similarity. IEEE Trans. Image Process..

[34] Zhang R., Isola P., Efros A.A., Shechtman E., Wang O. The unreasonable effectiveness of deep features as a perceptual metric. Proceedings of the IEEE Conference on Computer Vision and Pattern Recognition.

[35] Yue G., Zhang L., Zhang J., Xu Z., Wang S., Zhou T., Gong Y., Zhou W. (2024). Subjective quality assessment of thermal infrared images. Proceedings of the 2024 IEEE International Conference on Image Processing (ICIP).

[36] Zelmati O., Bondžulić B., Pavlović B., Tot I., Merrouche S. (2022). Study of subjective and objective quality assessment of infrared compressed images. J. Electr. Eng..

[37] Lee D.-G., Jeon M.-H., Cho Y., Kim A. (2023). Edge-guided multi-domain RGB-to-TIR image translation for training vision tasks with challenging labels. Proceedings of the 2023 IEEE International Conference on Robotics and Automation (ICRA).

[38] Panigrahi B., Kathala K.C.R., Sujatha M. (2023). A machine learning-based comparative approach to predict the crop yield using supervised learning with regression models. Procedia Comput. Sci..

[39] Cai W., Wei Z. (2020). PiiGAN: Generative adversarial networks for pluralistic image inpainting. IEEE Access.

[40] Bakurov I., Buzzelli M., Schettini R., Castelli M., Vanneschi L. (2022). Structural similarity index (SSIM) revisited: A data-driven approach. Expert Syst. Appl..

[41] Dosovitskiy A., Ros G., Codevilla F., Lopez A., Koltun V. CARLA: An open urban driving simulator. Proceedings of the Conference on Robot Learning. PMLR.

[42] Shaikh Z.A., Van Hamme D., Veelaert P., Philips W. (2022). Probabilistic fusion for pedestrian detection from thermal and colour images. Sensors.

[43] Dimitrievski M., Van Hamme D., Veelaert P., Philips W. (2020). Cooperative multi-sensor tracking of vulnerable road users in the presence of missing detections. Sensors.

[44] Chen T., Guestrin C. XGBoost: A Scalable Tree Boosting System. Proceedings of the 22nd ACM SIGKDD International Conference on Knowledge Discovery and Data Mining.

